# Fentanyl Plasma Concentrations after Application of a Transdermal Patch in Three Different Locations to Refine Postoperative Pain Management in Rabbits

**DOI:** 10.3390/ani10101778

**Published:** 2020-10-01

**Authors:** Valentina Mirschberger, Christian von Deimling, Anja Heider, Claudia Spadavecchia, Helene Rohrbach, Stephan Zeiter

**Affiliations:** 1 AO Research Institute Davos, 7270 Davos Platz, Switzerland; valentina_riehl@web.de (V.M.); deimling.ch@gmail.com (C.v.D.); 2 Swiss Institute of Allergy and Asthma Research (SIAF), University Zurich, 7265 Davos Wolfgang, Switzerland; anja.heider@siaf.uzh.ch; 3 Vetsuisse Faculty, Department for Veterinary Medicine, University of Bern, 3012 Bern, Switzerland; claudia.spadavecchia@vetsuisse.unibe.ch (C.S.); helene.rohrbach@vetsuisse.unibe.ch (H.R.)

**Keywords:** transdermal fentanyl patch, rabbit, postoperative analgesia, refinement

## Abstract

**Simple Summary:**

Fentanyl patches offer “stress free” postoperative pain management in rabbits. It has been shown that fentanyl uptake is dependent on exogenous and endogenous factors of the area where the patch is applied. The purpose of the study was to investigate three different locations (neck, inner and outer surface of the ear) to obtain reliable fentanyl plasma concentrations above those previously shown to be analgesic. The fentanyl plasma concentration was measured at several time points after patch application. In addition, the practicability of the proposed methods was evaluated. The group with application on the neck had the fastest uptake and equal to or over the analgesic plasma concentration for up to 72 h. The outer surface of the ear had slightly slower uptake and shorter analgesic duration whereas fentanyl uptake at the inner surface of the ear was insufficient to provide plasma analgesic concentration. The preparation of the neck proved to be the most laborious because of the thin and dense fur and the removal of the patch resulted in erythema. In conclusion, depending on how long potent analgesia is required, either the neck or the outer surface of the ear are suitable for patch application enabling “stress free” and reliable postoperative analgesia in rabbits.

**Abstract:**

Transdermal patches allow a noninvasive and “stress free” analgesia in rabbits. As fentanyl uptake is dependent on exogenous and endogenous factors of the area where the patch is applied, this study investigated three different locations (neck, inner and outer surfaces of the ear) for fentanyl patch application to provide adequate and reliable fentanyl plasma concentrations above those previously shown to be analgesic. Fentanyl plasma concentration was measured at different time points (3, 6, 9, 12, 18, 24, 36, 48, 72, 96, 120 h) and rabbits were assessed for their general conditions and treatment-related side effects. Practicability of the proposed methods was evaluated. Following patch application on the neck, fentanyl plasma concentrations equal to or above the analgesic value were measured in all rabbits between 6 and 72 h. Comparable concentrations were reached between 9 and 48 h in all animals for the outer ear surface. However, for the inner ear surface, analgesic concentrations were not reached, even if practicability was considered the best for this location. Preparation of the neck skin was judged as the most cumbersome due to the clipping of the dense fur and patch removal resulted in erythema. In summary, the application of the fentanyl patch on the neck and outer ear surface allowed the reach of reliable plasma concentrations above the analgesic threshold in rabbits. When applied on the neck, fentanyl patches provided the longest duration of analgesic plasma concentrations, whereas patch application and removal were easier on the outer ear surface.

## 1. Introduction

Rabbits are commonly used as animal models in biomedical research, e.g., orthopedic studies concerning bone healing, cartilage repair and infection studies [1,2,3]. In these experimental studies, the animals often undergo invasive surgeries which require systemic analgesia for several days. Looking into the literature, the administration of systemic analgesia in invasive experimental studies involving rabbits increased significantly from 16% (1995–997) to 50% (2005–2007) [4,5]. Additionally, despite improvement in the last 20 years it is reported that pain management is still inadequate for pet rabbits [6]. Nevertheless, optimization of postoperative analgesia protocols is still highly needed as studies investigating effective analgesia for surgical interventions have been rarely reported for this species [7]. Among opioids, the most commonly used drug was buprenorphine as the first-line therapy over this period [4].

Buprenorphine, a partial µ-receptor agonist opioid, is commonly used for perioperative pain control in laboratory animal species [4]. Despite the long duration of action, multiple injections are required daily to guarantee a continuous analgesic coverage [8]. As rabbits are highly susceptible to stress and anxiety [9,10], repeated handling and medical interventions should be kept to a minimum to improve animal welfare. As an alternative, titrating analgesic drugs to effect would carry the risk of letting pain go undertreated, as pain recognition in a prey species like the rabbit represents a true challenge [10,11], even though the rabbit grimace scale is a promising tool to assess postoperative pain [12].

An opportunity to ensure a noninvasive and “stress free” analgesia is through the use of transdermal patches which provide a continuous release of medications into the bloodstream over a considerable period. Fentanyl, a potent m-agonist synthetic opioid characterized by a short half time, would not be adequate for postoperative analgesia in rabbits as an injectable formulation, but becomes interesting as a transdermal patch. Fentanyl patches were developed by the Alza Corporation and came on the market in 1991. These patches provide a sustained and constant drug release into the systemic circulation for at least 72 h, and are commonly prescribed in human medicine to treat chronic cancer pain [13]. In 1995, the first animal study investigating these opioid releasing patches in dogs was published [14]. Later, their off-label use in the veterinary medicine, especially in the experimental fields for postoperative pain therapy, became popular due to their properties as a noninvasive long-term delivery form. In a study from Foley et al., the use of fentanyl patches placed on the neck of rabbits was investigated for the first time and considered to be efficacious [15]. In other species such as dogs, cats, sheep, horses and minipigs, the application of the analgesic patches has further been investigated [14,16,17,18,19,20,21,22,23]. It was shown that exogenous and endogenous factors such as the amount of subcutaneous fat, skin integrity, structure and arrangement of hair follicles, possible dermal depots, body core temperature, skin thickness, first-pass cutaneous biotransformation as well as the environmental temperature caused strong intra- and inter-individual differences in the uptake of fentanyl into the bloodstream. Therefore, the prediction of fentanyl plasma concentrations is difficult [5,8,14,18,24]. Based on this described variability of drug absorption depending on the application locations, it was hypothesized that there is a variation in the uptake of fentanyl into the blood when different application locations are used [24]. The goal of this study was to investigate different locations for fentanyl patch application (neck, inner and outer surfaces of the ear) in rabbits. We assumed that the application on the inner or outer surface of the ear would provide a more reliable absorption of fentanyl with less variability due to a more homogenous anatomy and easier site preparation.

## 2. Materials and Methods

### 2.1. Study Design

In this experimental, prospective study, the New Zealand White rabbits (NZW) were randomly and equally assigned to three groups, differing in the location of patch application: outer surface of the ear (group 1, n = 6), inner surface of the ear (group 2, n = 6) and neck between the scapulas (group 3, n = 6). In all groups, fentanyl patches were covered with a tape (Leukoplast, BSN Medical GmbH, Hamburg, Germany) to ensure a continuous contact throughout the study period.

**Figure 1:**
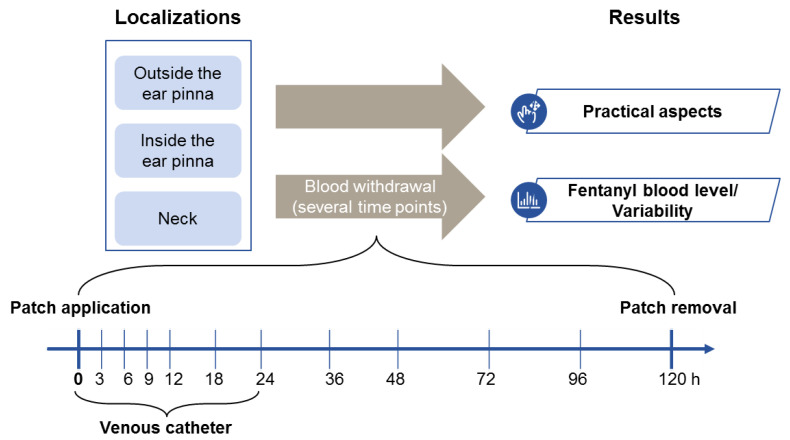
Study design. Fentanyl plasma concentrations higher than 0.5 ng/mL were considered to be analgesic based on previous reports [13,19]. Outcome parameters were time spent above threshold concentration, variability of fentanyl plasma concentrations and practicability of patch application.

Venous blood samples withdrawn at different time points (Figure 1) were centrifuged and frozen at −80 °C until analysis of fentanyl plasma concentration. Additionally, treatment-related side effects were recorded, and practicability of the proposed methods was evaluated to determine the most adequate location for patch application in rabbits. No surgical procedures were performed as only the drug’s absorption curve was investigated in this trial.

### 2.2. Animals

Eighteen specific pathogen free (according to FELASA guidelines), female New Zealand White rabbits, weighing 3.05 to 3.80 kg with an age of 23 to 24 weeks at the start of the study were purchased from Charles River Laboratories (Sulzfeld, Germany). The study was performed in an AAALAC (Association for Assessment and Accreditation of Laboratory Animal Care) approved institution according to the Swiss animal welfare regulations and approved by the ethical committee of the canton Graubünden, Switzerland (No. 2016_40).

Upon delivery, the animals were given at least two weeks to acclimatize to the housing conditions (17 +/− 2 °C, > 30% humidity, 10 to 20 air changes per hour 12:12 h light-dark cycle). Animals were group housed with a maximum of 15 animals per group, on straw and fed with hay, supplemental food (3140, Kaninchen & Meerschweinchen, Haltung, Standard, Kliba NAFAG, Switzerland) as well as some fresh carrots daily. Animals had free access to water. For the 5–day study duration, rabbits were single housed in stainless steel cages with ground floor area of 5600 cm^2^. The cages are 62 cm high and there is an increase area with an area of 1971 cm^2^, underneath which the rabbits can hide. The cages were littered down with straw. Feeding was done the same manner as before, providing a piece of wood for gnawing and offering the hay in a fodder rack as additional environmental enrichment. Before inclusion into the study, the rabbits underwent a general clinical examination by a veterinarian. During the experimental period, the animals’ welfare was regularly evaluated at least twice a day by a veterinarian. After the end of the study, the rabbits were reintegrated in their initial group.

### 2.3. Materials

In this study, a 12 µg/h transdermal fentanyl patch (Fentanyl-Mepha Matrixpfl, Mepha Pharma AG, Switzerland) was used. Due to the recommendation of a dosage of 2 µg/kg/h in sheep and up to 5 µg/kg/h in small animals (dogs, cats) described in several former studies, the 12 µg/h patch size was chosen [5,7,22]. The mean administered dose rate of fentanyl was 3.60 µg/kg/h (Dmax = 3.93 µg/kg/h, Dmin = 3.16 µg/kg/h).

### 2.4. Fentanyl Patch Application Process

Rabbits were sedated with Medetomidin (200 μg/kg) and Midazolam (0.5 mg/kg) both mixed in a syringe and given intramuscularly approximately 15 min before starting skin preparation. Thereafter, the rabbits were placed in sternal recumbency, eye ointment (Vitamin A Blache Augensalbe, Bausch & Lomb Swiss AG, Switzerland) was applied and oxygen (flow rate 1 L/min) was provided via face mask. The required site was carefully clipped using two different clippers depending on the location. For the location on the ear, the clipper Isis (GT420 Aesculap, Germany; cutting length 0.5 mm) was utilized. For the neck, another mechanical fur clipper (model GH703/10 (Favorita II GT104), Aesculap, Germany; cutting length 0.1 mm) was used in the interscapular region due to the increased density of hair at this site. During clipping extreme precautions were taken not to traumatize the application site to avoid influencing fentanyl absorption.

Afterwards, skin was degreased with swabs (Mesoft, Moelnlycke, Sweden) soaked in alcohol (Softasept, B. Braun Vet Care GmbH, Germany) to ensure a good contact of the patch with the skin. While waiting for 5 min to air dry, the ear not used for patch application was clipped and prepared aseptically with alcohol for the placement of a 22 G catheter (Vasofix Safety, B.Braun Meisungen AG, Germany) in the marginal ear vein. The catheter, inserted to facilitate blood withdrawal, was wrapped with tape (Durapore, 3 M (Schweiz) GmbH, Switzerland) and a roll of gauze (Mesoft) to hold it in place. After each blood withdrawal, the catheter was flushed with approximately 0.5 mL of heparinized 0.9% NaCl (B.Braun Meisungen AG, Germany) and a mandrin (Mandrin Vasofix, B.Braun Meisungen AG, Germany) was inserted. On the dry application site, the sticky side of the patch was then pressed on the skin with the palm of the hand and kept there for 1 min to ensure a good patch-skin contact. Finally, the patch was fixed with tape (Leukoplast) to prevent the loosening of the patch detachment during animal handling.

### 2.5. Sample Collection and Plasma Fentanyl Analysis via ELISA

Venous blood sampling was performed immediately prior to the application of the patch and 3, 6, 9, 12, 18, 24, 36, 48, 72, 96, 120 h thereafter. In a few cases arterial blood was sampled, if no venous sample could be obtained. At each sampling time point, approximately 1 mL of blood was collected in a 1.3 mL EDTA-covered tube (1.3 mL K3E, Sarstedt, AG & Co, Nümbrecht, Germany). A venous catheter was always used to withdraw blood samples. For the first 24 h the catheter was left in the marginal ear vein. Thereafter, a new catheter was inserted for each blood sampling. EDTA-tubes were then centrifuged for 15 min, at 23 °C and 1000 rcf (Centrifuge 5810R, Vaudaux-Eppendorf AG, Switzerland) within 2 h of collection.

Subsequently, the obtained plasma was transferred into Eppendorf tubes (Vaudaux-Eppendorf AG, Switzerland) and stored at −80 °C until analysis. Plasma fentanyl concentrations were measured by using a commercially available human enzyme-linked immunoabsorbent assay (ELISA). Fentanyl Kits (Adnova, Fentanyl (Human) ELISA Kit, Taiwan) were used according to the manufacturer’s instructions. Additionally, serial dilutions of fentanyl standard (Fentanyl Sintetica 0.5 mg/10 mL) in fentanyl negative rabbit plasma were measured on the ELISA-plate, as a standard curve. The absorbance was read at 450 nm using a Mithras microplate reader (Berthold Technologies, Bad Wildbad, Germany). Each sample was tested in duplicates.

### 2.6. Scoring and Practicability

During the study, the rabbits were scored twice a day. The scoring system included observation of food and water intake, assessment of the general conditions, body weight and rectal temperature as well as defecation and coprophagy. Additionally, practicability was assessed based on the following criteria: ease of preparation, quality of patch adhesiveness, ease of daily checks, occurrence of undesired patch detachment before the end of the study, ease of patch removal and skin condition after patch removal. Any findings in respect to these criteria were noted and at the end a subjective three level scoring system (positive, neutral and negative assessment) comparing the three groups was applied by one veterinarian.

### 2.7. Statistical Analysis

For all locations descriptive statistics was used to describe fentanyl uptake over time. The goal of this study was not to find significant difference between groups. As we are aiming towards a reliable postoperative analgesia for all animals, the main outcome was the time all animals of one group had a fentanyl plasma concentration above the threshold of 0.5 ng/mL.

## 3. Results

One rabbit was excluded from the study and subsequently replaced due to CNS symptoms occurring 18 h after patch application. Necropsy revealed a thromboembolism as the most likely reason for the symptoms, caused by repeated blood withdrawal.

### 3.1. Plasma Fentanyl Concentrations

**Figure 2:**
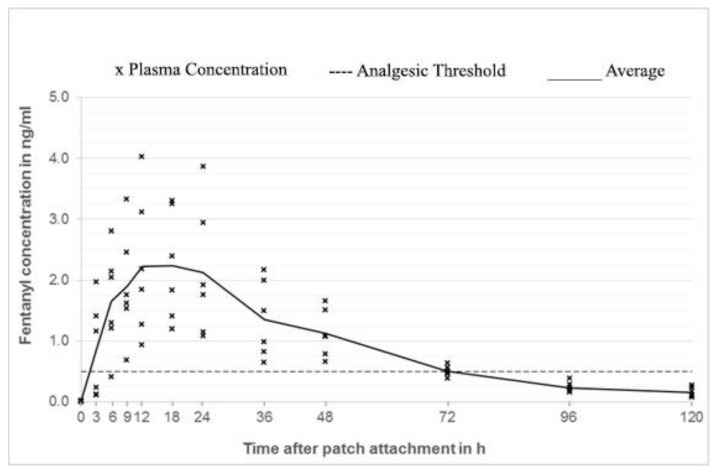
Fentanyl plasma concentrations group 1 (outer surface of the ear).

**Figure 3:**
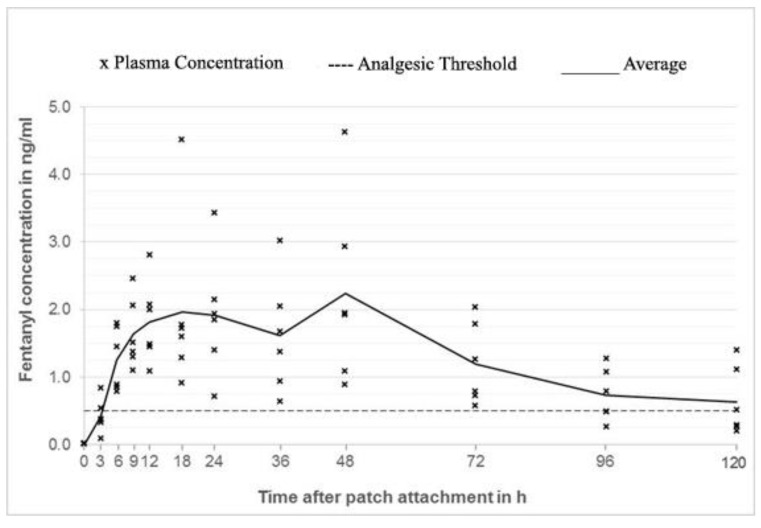
Fentanyl plasma concentrations group 3 (neck).

**Figure 4:**
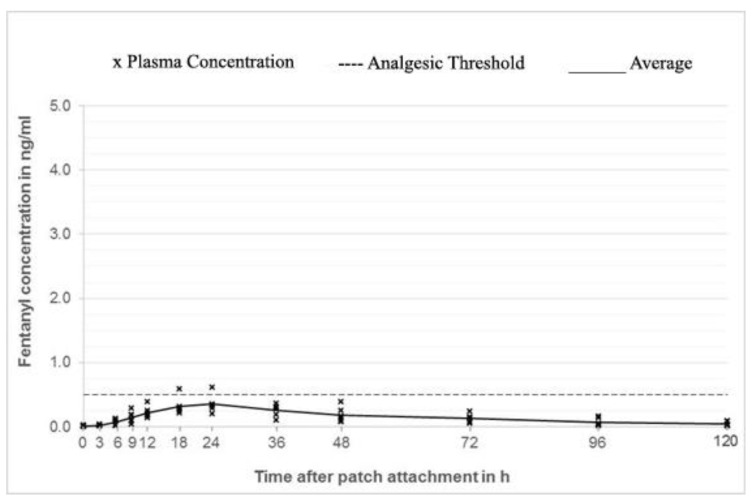
Fentanyl plasma concentrations group 2 (inner surface of the ear).

In group 1 (outer surface of the ear; Figure 2) plasma concentrations considered to be analgesic (0.5 ng/mL) was reached in 3 out of 6 rabbits 3 h after patch application, while all the animals in this group were above threshold at 9 h. In group 3 (neck; Figure 3), 2 out of 6 animals reached threshold at 3 h, while all 6 animals at 6 h (mean plasma concentration of 1.26 ng/mL). In contrast, only 1 out of 6 rabbits in group 2 (inner surface of the ear; Figure 4) reached the threshold at 18 h, while the others stayed below the threshold for the whole duration of the study.

**Figure 5:**
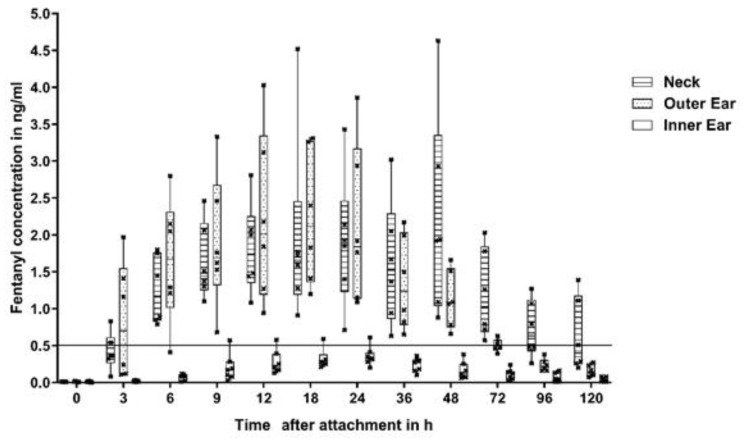
Fentanyl plasma concentrations of all groups.

Mean steady state concentrations in group 1 and 3 were similar (2.24 and 2.23 ng/mL) but the timing was different. Group 1 reached the peak concentration at 18 h, whereas group 3 at 48 h. Every animal of group 1 reached concentrations above 0.5 ng/mL between 9 and 48 h, while animals of group 3 between 6 and 72 h after patch application (Figure 5).

### 3.2. Practicability

Findings of the practicability assessment are summarized in Table 1.

*Ease of preparation:* For patch application on the outer surface of the ear, group 1 needed a moderate effort to clip and disinfect the skin. Compared to the outer surface of the ear, the inner surface (group 2) needed minimal effort to clip due to almost no hair growth. However, it took longer to degrease the skin. Group 3, with the patch located on the neck, showed the highest density of hair growth combined with very thin hair. As a result, even with another more powerful clipper, it took longer to clip the fur in this location. Additionally, fighting wounds were discovered in this location while clipping, which made it hard to place the patch in exactly the same position on each rabbit avoiding these skin lesions.

*Quality of patch adhesiveness* was comparable in all groups—no difference could be noticed.

*Ease of daily checks:* Animals of group 1 were the easiest to check, as the outer surface of the ear was promptly visualized. Tape loosening could be seen without manipulation. In contrast, in group 2 loosening of the tape and patch underneath could only be noticed while handling the rabbit. Group 3 differed from the other groups as a check of the patch was only feasible while holding the rabbit, as the patch on the neck resulted covered by the surrounding fur.

*Occurrence of undesired patch detachment:* Patch loosening in terms of detaching edges occurred starting 48 h after patch application particularly in group 2 and 3 but also in one animal of group 1, 72 h after patch application. Signs of mild manipulation (scratching) of the tape by the rabbits could be observed in all groups suggesting some irritation engendered by the presence of the tape/ patch. While in group 1 manipulation lead to partial patch loosening in just one animal, detaching of the patches occurred in 5 of 6 animals of group 2 and 3 of 6 in group 3. Obvious and strong manipulation of the tape covering the patch was visible in 2 of 18 animals namely in group 2 and 3.

*Ease of patch removal:* Patch removal 120 h after application was the easiest and most stress-free for the animals in group 2 due to greasy skin and just a few regrown hair at this location. In contrast, group 1 and 3 showed a high density of regrown fur, which made it painful and hard to remove the tape and the patch in all these animals.

**Figure 6:**
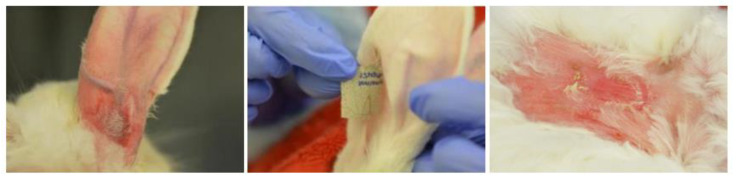
Representative images of skin condition after patch removal (**left**: group 1, **middle**: group 2 and **right**: group 3).

*Skin condition after patch removal:* Group 2 showed no signs of skin reaction after patch removal, whereas in group 1 and 3, a diffuse erythema underlying the drug-delivery portion of the patches was recognized in all the animals (Figure 6).

No differences in general condition, food or water intake, body weight, defecation, coprophagy or temperature were noticed among the three groups.

## 4. Discussion

The aim of the study was to determine fentanyl plasma concentrations in rabbits from patches applied at three different locations with the final aim to refine (postoperative) analgesia. A standard skin preparation and patch application protocol was applied to avoid inconsistent results as previously reported [16]. For the different locations, fentanyl plasma concentrations were measured, and practicability of patch application, maintenance and removal were assessed. To the authors’ knowledge, the current study is the first to report fentanyl plasma concentrations in rabbits following patch application at different locations.

The results of this study indicate that the patch placed on the neck provided fentanyl plasma concentrations higher than 0.5 ng/mL from 6 to 72 h after application. This plasma concentration was reached earlier and peak concentrations were higher when the patch was applied on the neck compared to both ear sites. However, preparation of this location was the most complex due to the high density of fur. Additionally, animal handling was needed to check the patch adhesion on the skin. Furthermore, patch removal was accompanied by aversive reactions and lead to mild skin erythema. The cause of the erythema associated with patch application seemed to be due to the occlusive nature of the patch systems [24]. Cutaneous irritation was often encountered and limits the duration of time, a patch can be worn at a single site. However, the adhesive agent on the tape may be causally related, too.

For patches applied on the outer ear surface analgesic plasma fentanyl concentrations were measured from 9 to 48 h, whereas skin preparation was easier than in group 3. We hypothesize, that a reason for the difference in fentanyl absorption might be owed to the influence of a difference in body temperature, as it is described in several former studies. In fact, a temperature of 40 °C can raise drug delivery up to one third [8,25]. Rabbits can regulate their ear blood flow, which might lead to a lower drug delivery due to a lower body temperature in contrast to the neck. Depending on how long potent analgesia is requested, the application on the outer surface of the ear pinna might still be relevant for the attachment of fentanyl patches. There is a study published by Christou et al. using sheep, in which the change of fentanyl patches after 24 and 72 h is performed to investigate the fentanyl plasma concentrations thereafter [26]. It was shown that a patch change lead to an extended period of analgesia without a lack of insufficient pain management in between and a higher peak concentration due to pre-loading. Additionally, the peak concentration increased in that study, although we assume that this does not represent an advantage, as long as a certain threshold plasma concentration is exceeded permanently. Most likely the incidence of side effects will increase with rising values.

An obvious finding of our study is that it is inappropriate to apply the fentanyl patch on the inner surface of the ear. While practicability was good and the upcoming burden for the rabbit low, fentanyl plasma concentrations were below the predefined threshold, which implies the unsuitability of this location. Low plasma concentrations in this group might be attributable to poor drug penetration due to endogenous and exogenous influences such as differing skin conditions as described in former studies [24,27]. Additionally, rate of manipulation was higher in this group, which probably favored detaching of the patches in 5 of 6 animals. Furthermore, fixation of the patch with tape was more difficult due to the concave anatomy of the ear pinna, which probably also facilitated detachment. However, frequent checks to ensure that the patch is still in place were necessary in all groups.

Individual peak values of animals of group 1 and 3 showed a high variability within the study period. Probably several individual factors, like different pH, variable depot formation in the stratum corneum and dermis, different body temperatures and differences in cutaneous blood flow, influenced this outcome as previously reported [24].

The neck has been already described for patch application in rabbits, but a detailed description of skin preparation and patch application process have not been reported [15]. In the present study, the method of application was accurately planned and described to avoid variations in drug absorption due to differences in site preparation. Riviere and Papich showed that abrasion removes the stratum corneum barrier and can alter the transdermal flux [24]. Foley and coworkers investigated plasma fentanyl concentrations in rabbits using different methods of hair removal. For rabbits with clipped hair, comparable to the study described here, they reported a peak of 1.11 ± 0.32 ng/mL at 24 h and a decrease to 0.77 ± 0.21 ng/mL 72 h after patch application [15]. This is lower than the peak values in this study, even though fentanyl patches with a half dose rate were used: 3.60 µg/kg/h in this study versus 6.67 µg/kg/h reported by Foley and coworkers. One possible explanation for the better uptake is the use of alcohol to degrease the skin and ameliorate the drug uptake in this study. Two other studies reported the use of alcohol to degrease the skin prior to patch application in sheep [8,26]. In other studies involving dogs and cats, no degreasing with alcohol was performed [18,28,29].

The main limitation of this study is that rabbit-specific fentanyl plasma concentration able to provide analgesia is unknown. In humans, the minimum effective plasma concentration for analgesia is assumed to range between 0.5 and 2.0 ng/mL [11]. Previous veterinary studies extrapolated this range to animals such as sheep, rabbits and minipigs [15,16,23]. In former human studies, wide ranges of 0.23 to 3 ng/mL were described [13,30]. In humans, contrasting evidence has been reported about the relationship between respiratory adverse effects and fentanyl plasma concentration. While in one study concentrations above 2.0 ng/mL were suggested to cause respiratory depression [31]. In another study no correlation between fentanyl plasma concentrations and side effects was found [32]. In our study, no obvious adverse effects were observed, although concentrations above 2.0 ng/mL were measured. However, there was no evaluation of heart rate and respiratory frequency, which are known among others to be influenced at an early stage [33]. In cats, a plasma concentration higher than 2.2 ng/mL was observed to cause dysphoric behavior [17]. No dysphoria was observed in the rabbits of the present study.

Further limitations of this study are the use of only female rabbits. In humans, no difference in fentanyl uptake between male and female patients has been shown. To our knowledge, this has not been investigated yet in animals [34]. Another limitation is the use of a commercially available enzyme-linked immunosorbent assay (ELISA) developed to analyze human samples and no second test was performed to verify the results. In the literature the use of several different tests for analysis of fentanyl plasma concentration has been reported. A human ELISA was used in sheep, while no details were provided about the ELISA method applied in the study by Gilbert et al. [16,26]. In contrast to this, tests as the liquid chromatography–MS and gas chromatography–MS methods served for analysis in humans as well as in sheep [16,30,35]. Besides these tests, radioimmunoassays (RIA) are commonly used for analysis of plasma or serum fentanyl concentrations, too [29,36].

Concerning the timing of fentanyl patch application in experimental settings, some additional aspects need to be considered. Based on the findings of the present study, at least 6 h are needed for the fentanyl patch to provide a potentially analgesic plasma concentration. This implies that additional analgesics are needed to cover this period if the patch is applied during preparation for surgery. Some authors recommend patch application 12–24 h prior to surgery in dogs and sheep to achieve sufficient analgesia for surgery [7,12,37]. However, earlier application also means more stress for the rabbit, as an additional handling session accompanied by sedation would be needed. Another important aspect is the desired duration of pain treatment with opioids, which will depend on the invasiveness of surgery as well as administration of other pain medication (e.g., NSAIDs). This has to be defined for each study or patient and adapted as needed. Patches can be removed earlier or a second patch could be applied to extend the analgesic effect. As already mentioned, the effects of a patch change were investigated in sheep [26], while no comparable studies have been performed in rabbits so far.

## 5. Conclusions

The present findings support the use of transdermal fentanyl patches as a method to provide long-lasting analgesia in NZW rabbits. Depending on how long potent analgesia is required, either the neck or the outer surface of the ear are suitable for patch application. Summarizing, the administration of the patch can simplify postoperative pain management in laboratory rabbits and improve animal welfare in sense of the 3R.

## Figures and Tables

**Table 1:** Assessment of the practicability for the different locations. For each criteria, a subjective three level scoring system (positive, neutral and negative assessment) comparing the three groups was applied.

Aspect	Group 1 (Outside Ear)	Group 2 (Inside Ear)	Group 3 (Neck)
Ease of preparation	Neutral	Positive	Negative
Quality of patch adhesiveness	Positive	Positive	Positive
Ease of daily checks	Positive	Neutral	Negative
Occurrence of undesired patch detachment	Positive	Negative	Neutral
Ease of patch removal	Negative	Positive	Negative
Skin condition after patch removal	Negative	Positive	Negative
